# Comparison of Use of the Massachusetts Child Psychiatry Access Program and Patient Characteristics Before vs During the COVID-19 Pandemic

**DOI:** 10.1001/jamanetworkopen.2021.46618

**Published:** 2022-02-02

**Authors:** Yael Dvir, Clare Ryan, John H. Straus, Barry Sarvet, Ireen Ahmed, Kathryn Gilstad-Hayden

**Affiliations:** 1 Division of Child and Adolescent Psychiatry, Department of Psychiatry, University of Massachusetts Chan Medical School, UMass Memorial Medical Center, Worcester; 2 University of Massachusetts Medical School, Worcester; 3 Massachusetts Child Psychiatry Access Program/Beacon Health Options, Boston; 4 Department of Psychiatry, University of Massachusetts-Baystate, Springfield; 5 Division of Child and Adolescent Psychiatry, Department of Psychiatry, Tufts Medical Center, Boston, Massachusetts; 6 Department of Psychiatry, Yale School of Medicine, New Haven, Connecticut

## Abstract

This cross-sectional study compares the number of encounters at the Massachusetts Child Psychiatry Access Program, patient characteristics, and mental health diagnoses before vs during the COVID-19 pandemic.

## Introduction

COVID-19 pandemic–related social isolation, school closures, and anxiety about the future have been negatively associated with youths’ mental health.^1^ During the pandemic, increased depression and anxiety and an increased proportion of emergency department visits for mental health concerns, including suicide, have been demonstrated.^2,3^ With scarce mental health resources for youths,^4^ pediatric primary care clinicians (PCCs) play a critical role in behavioral health treatment and referrals. Published data about the mental health effects of the COVID-19 pandemic do not capture the substantial number of youths who do not visit the hospital. Thus, we examined data from the Massachusetts Child Psychiatry Access Program (MCPAP), established in 2004 to aid PCCs in providing psychiatric treatment through consultation and referral services.^5^

## Methods

For this cross-sectional study, we collected data from the MCPAP database for counts of unique patient telephone and in-person encounters during fiscal years (FYs) 2019 through 2021. The UMass Chan Medical School institutional review board indicated that oversight and informed consent was not required because the study team did not access private identifiable information. This study followed the Strengthening the Reporting of Observational Studies in Epidemiology (STROBE) reporting guideline.

The number of monthly encounters in FY 2021 was compared with those in FY 2019 and FY 2020 using a Mann-Whitney *U* test. Using available data, we conducted χ^2^ tests to compare the counts of encounters before the COVID-19 pandemic (March and April in 2018 and 2019) and during the pandemic (March and April in 2021) by sex, type of insurance, and patient age. These months were chosen for consistency relative to the timing of the pandemic and to minimize seasonal differences. The mean number of monthly encounters by mental health diagnosis was compared between periods before and during the pandemic using descriptive data (means and percentage change). *P* < .05 using a 2-tailed test was considered statistically significant. Analyses were conducted using SAS, version 9.4 (SAS Institute, Inc).

## Results

This study included 2515 unique patients with encounters at the MCPAP before the COVID-19 pandemic and 1700 unique patients with encounters during the pandemic. Patient characteristics are presented in the Table. During the pandemic, patients were more likely to be female compared with before the pandemic (904 [54%] vs 1102 [44%]; odds ratio, 1.47 [95% CI, 1.30-1.67) and were less likely to be aged 12 years or younger (657 [39%] vs 1283 [51%]; odds ratio, 0.60 [95% CI, 0.53-0.68]). No difference in the percentage of patients with private insurance during and before the pandemic was found (898 [58%] vs 1252 [55%]; odds ratio, 1.10 [95% CI, 0.97-1.26]). The mean number of unique patient encounters per month increased for each mental health diagnosis from before to during the pandemic: anxiety, 211 to 301; depression, 166 to 238; autism spectrum disorder, 31 to 82; attention-deficit/hyperactivity disorder, 172 to 175; and other, 264 to 307. The greatest percentage increase was for encounters for autism spectrum disorder (165%), followed by anxiety (43%), depression (43%), other (16%), and attention-deficit/hyperactivity disorder (2%).

**Table.** Characteristics of Patients With Encounters at the Massachusetts Child Psychiatry Access Program Before vs During the COVID-19 Pandemic

Characteristic	Patients, No. (%)	
	March and April of 2018 and 2019 (n = 2515)	March and April of 2021 (n = 1700)
Sex^a^		
Female	1102 (44)	904 (54)
Male	1406 (56)	783 (46)
Insurance		
Private	1252 (55)	898 (58)
Public	1018 (45)	662 (42)
Unknown or none	245	140
Age, y^b^		
0-5	178 (7)	77 (5)
6-12	1105 (44)	580 (34)
13-18	1086 (44)	864 (51)
>18	129 (5)	168 (10)

^a^ Column sums do not equal the total number of patients because some patients did not identify as male or female.

^b^ Column sums do not equal the total number of patients owing to missing data.

The Figure shows the number of unique patient monthly encounters during FYs 2019 through 2021. A Mann-Whitney *U* test indicated that the number of monthly encounters was greater in FY 2021 (median, 670.5; range, 429.0-925.0) compared with FY 2019 (median, 538.5; range, 413.0-715.0; *U* = 38; *P* = .054) and FY 2020 (median, 516.0; range, 388.0-645.0; *U* = 26; *P* = .009).

**Figure.**
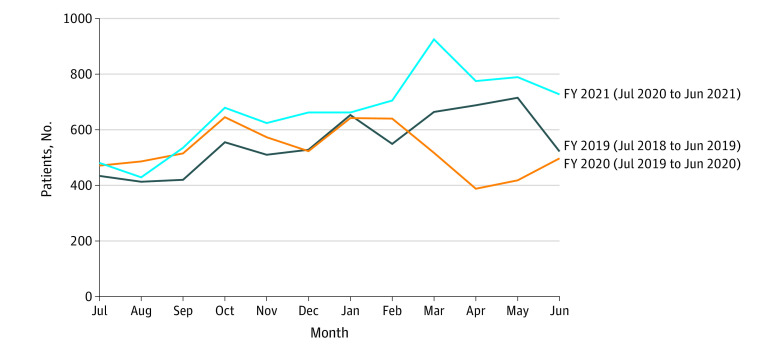
Unique Patients Served per Month at the Massachusetts Child Psychiatry Access Program During Fiscal Years (FYs) 2019 Through 2021

## Discussion

The decrease in MCPAP use during the early months of the COVID-19 pandemic found in this study corresponds with the overall initial decrease in health care use not directly associated with COVID-19.^6^ Data from March and April of 2021 showed increased health care use compared with March and April 2018 and 2019, particularly consultations for anxiety and depression, aligning with published data.^1^ Of note, the diagnosis with the greatest increase in MCPAP consultations was autism spectrum disorder. We hypothesize that decreased availability of in-home applied behavioral analysis and in-person special education was associated with disruption and stress for youths and families who depend on them. Girls and older adolescents were more likely to present to their PCCs, but the structure of our data did not allow us to explore interactions between sex, age, and type of presenting condition, limiting the interpretation of the results.

Our data suggest a trend of increasing need for pediatric behavioral health services and treatment on an outpatient basis. Although the data cannot suggest a cause for increased needs among youths, in our study, the number of youths presenting to their PCCs with mental health concerns and the complexity of presentation increased during the pandemic compared with before the pandemic. This finding suggests a need for increasing capacity to meet demand.
